# Selective Estrogen Receptor β Agonists: a Therapeutic Approach for HIV-1 Associated Neurocognitive Disorders

**DOI:** 10.1007/s11481-019-09900-y

**Published:** 2019-12-19

**Authors:** Kristen A. McLaurin, Landhing M. Moran, Rosemarie M. Booze, Charles F. Mactutus

**Affiliations:** Program in Behavioral Neuroscience, Department of Psychology, University of South Carolina, 1512 Pendleton Street, Columbia, SC 29208 USA

**Keywords:** S-Equol, Preattentive processes, Sustained attention, Selective attention

## Abstract

The persistence of HIV-1 associated neurocognitive disorders (HAND) in the post-cART era, afflicting between 40 and 70% of HIV-1 seropositive individuals, supports a critical need for the development of adjunctive therapeutic treatments. Selective estrogen receptor β agonists, including S-Equol (SE), have been implicated as potential therapeutic targets for the treatment of neurocognitive disorders. In the present study, the therapeutic efficacy of 0.2 mg SE for the treatment of HAND was assessed to address two key questions in the HIV-1 transgenic (Tg) rat. First, does SE exhibit robust therapeutic efficacy when treatment is initiated relatively early (i.e., between 2 and 3 months of age) in the course of viral protein exposure? Second, does the therapeutic utility of SE generalize across multiple neurocognitive domains? Treatment with SE enhanced preattentive processes and stimulus-response learning to the level of controls in all (i.e., 100%) HIV-1 Tg animals. For sustained and selective attention, statistically significant effects were not observed in the overall analyses (Control: Placebo, *n* = 10, SE, *n* = 10; HIV-1 Tg: Placebo, *n* = 10, SE, *n* = 10). However, given our a priori hypothesis, subsequent analyses were conducted, revealing enhanced sustained and selective attention, approximating controls, in a subset (i.e., 50%, *n* = 5 and 80%, *n* = 8, respectively) of HIV-1 Tg animals treated with SE. Thus, the therapeutic efficacy of SE is greater when treatment is initiated relatively early in the course of viral protein exposure and generalizes across neurocognitive domains, supporting an adjunctive therapeutic for HAND in the post-cART era.

**Graphical Abstract:**
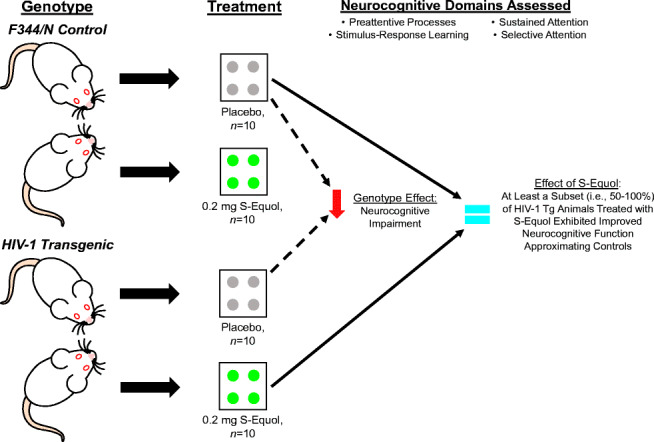
HIV-1 transgenic (Tg) and control animals were treated with either 0.2 mg S-Equol (SE) or placebo between 2 and 3 months of age (Control: Placebo, *n* = 10, SE, *n* = 10; HIV-1 Tg: Placebo, *n* = 10, SE, *n* = 10). Neurocognitive assessments, tapping preattentive processes, stimulus response learning, sustained attention and selective attention, were conducted to evaluate the utility of SE as a therapeutic for HIV-1 associated neurocognitive disorders (HAND). Planned comparisons between HIV-1 Tg and control animals treated with placebo were utilized to establish a genotype effect, revealing prominent neurocognitive impairments (NCI) in the HIV-1 Tg rat across all domains. Furthermore, to establish the utility of SE, HIV-1 Tg animals treated with SE were compared to control animals treated with placebo. Treatment with 0.2 mg SE ameliorated NCI, to levels that were indistinguishable from controls, in at least a subset (i.e., 50–100%) of HIV-1 Tg animals. Thus, SE supports an efficacious, adjunctive therapeutic for HAND.

HIV-1 transgenic (Tg) and control animals were treated with either 0.2 mg S-Equol (SE) or placebo between 2 and 3 months of age (Control: Placebo, *n* = 10, SE, *n* = 10; HIV-1 Tg: Placebo, *n* = 10, SE, *n* = 10). Neurocognitive assessments, tapping preattentive processes, stimulus response learning, sustained attention and selective attention, were conducted to evaluate the utility of SE as a therapeutic for HIV-1 associated neurocognitive disorders (HAND). Planned comparisons between HIV-1 Tg and control animals treated with placebo were utilized to establish a genotype effect, revealing prominent neurocognitive impairments (NCI) in the HIV-1 Tg rat across all domains. Furthermore, to establish the utility of SE, HIV-1 Tg animals treated with SE were compared to control animals treated with placebo. Treatment with 0.2 mg SE ameliorated NCI, to levels that were indistinguishable from controls, in at least a subset (i.e., 50–100%) of HIV-1 Tg animals. Thus, SE supports an efficacious, adjunctive therapeutic for HAND.

## Introduction

The advent of combination antiretroviral therapy (cART), the primary treatment regimen for individuals with human immunodeficiency virus type 1 (HIV-1), dramatically decreased the severity of neurocognitive deficits associated with HIV-1 (Ances and Ellis 2007). However, HIV-1 associated neurocognitive disorders (HAND) persist, afflicting between 40 and 70% of HIV-1 seropositive individuals (Letendre et al. 2010; McArthur et al. 2010; Heaton et al. 2011). In the post-cART era, HAND has been defined as a progressive, neurodegenerative disease (Cohen et al. 2015; McLaurin et al. 2019a) characterized by alterations in speed of information processing, attention, working memory, and executive function (e.g., Cysique et al. 2004; Garvey et al. 2009; Heaton et al. 2011). Due to the prevalence of HAND in the post-cART era, and its progressive nature (Heaton et al. 2015; Gott et al. 2017; McLaurin et al. 2019a), there is a critical need to develop additional neuroprotective and/or neurorestorative therapeutics.

Estrogen receptors (ER), which belong to the nuclear receptor family of transcription factors, are classified into two primary subtypes, including ERα (Jensen 1962), and ERβ (Kuiper et al. 1996). Although both ERα and ERβ bind to 17β-estradiol with high affinity (Kuiper et al. 1996) and share structural characteristics (e.g., near-identical DNA-binding domain (96%), Kuiper et al. 1996), significant differences in tissue distribution and biological effects have been observed (e.g., Kuiper et al. 1997). Specifically, ERα is predominant in reproductive organs (e.g., uterus, mammary glands), skeletal muscle and bone, playing a critical role in maintaining female reproductive functions (for review, Paterni et al. 2014). ERβ, however, is involved in mediating estradiol signaling in the immune and central nervous systems (for review, Paterni et al. 2014). Within the central nervous system, cells containing ERβ mRNA or immunoactivity are widely dispersed (e.g., Li et al. 1997; Shughrue et al. 1997; Zhang et al. 2002; Gonzalez et al. 2007), and observed in brain regions (e.g., prefrontal cortex, ventral tegmental area, hippocampus) commonly associated with HAND (e.g., Maki et al. 2009; Israel et al. 2019).

Since 2005 (Kendall et al. 2005; Wallace et al. 2006), multiple studies have been conducted to evaluate the utility of estrogenic compounds to protect against the neurotoxic effects of HIV-1 viral proteins. Initial in vitro studies reported that pretreatment with ER agonists blocked neurotoxic effects (Kendall et al. 2005), attenuated oxidative stress (Wallace et al. 2006) and prevented the loss of dopamine transporter function (Wallace et al. 2006) induced by HIV-1 viral proteins (i.e., Tat, gp120). Subsequent investigations were targeted at evaluating whether the neuroprotective effects of estrogen occurred via an ER sensitive or non-receptor mediated mechanism; studies which revealed that the ERβ subtype mediated the 17β-estradiol attenuation of Tat-induced apoptotic signaling in cortical cell cultures (Adams et al. 2010). ERβ, therefore, may support a key target for the development of adjunctive therapeutics for HAND in the post-cART era.

Phytoestrogens, which exhibit a higher affinity for ERβ than ERα (e.g., Kuiper et al. 1998; Mueller et al. 2004), are plant-derived compounds that are structurally similar to 17β-estradiol (Glazier and Bowman 2001). Isoflavones, including genistein, daidzein (DAI), and glycitein, are one class of phytoestrogens commonly found in soy products (Murphy et al. 1982; Setchell 1998). Equol is an active metabolite produced by gut microbiota following the ingestion of the soy derived phytoestrogen DAI (Setchell et al. 1984). S-Equol (SE), the only enantiomer produced by humans (Setchell et al. 2005), exhibits neuroprotective effects via its selective affinity for ERβ (Setchell et al. 2005; Bertrand et al. 2015). Furthermore, when SE crosses the blood-brain-barrier it distributes most significantly to the prefrontal cortex (Lund et al. 2001); a brain region associated with higher-order cognitive functioning. Most critically, however, the translational relevance of SE is evidenced by its progression into clinical trials for Alzheimer’s disease (Ausio Pharmaceuticls; NCT03101085), another progressive, neurodegenerative disorder.

SE has been implicated as a potential adjunctive therapeutic for HAND in both in vitro (Bertrand et al. 2015) and in vivo (Moran et al. 2019) studies. In primary neuronal cell cultures, pretreatment with SE prevented synapse loss induced by the HIV-1 viral protein, Tat (Bertrand et al. 2015). Precursors to SE, including DAI and liquiritigenin (LQ), which also selectively target ERβ (DAI: Casanova et al. 1999; LQ: Mersereau et al. 2008), also prevented Tat induced neuronal apoptosis (Adams et al. 2012) and restored synaptodendritic injury (Bertrand et al. 2014). Given the utility of phytoestrogens to prevent and restore synaptic function in vitro, subsequent in vivo studies were targeted at protecting and/or restoring neurocognitive function following constitutive expression of HIV-1 viral proteins; a therapeutic approach that may be key to effectively treating neurocognitive deficits in HAND. In a dose-response study in the HIV-1 transgenic (Tg) rat, treatment with SE between 6 and 8 months of age enhanced sustained attention, to the level of controls, in a subset (i.e., 40%) of animals (Moran et al. 2019). To date, however, the generalizability of the therapeutic efficacy of SE when treatment occurs at an earlier age and across neurocognitive domains has not yet been assessed.

Neurocognitive functions, including preattentive processes, stimulus-response learning, and attention, are generally componential (Keeler and Robbins 2011). Specifically, sensory input is transformed to motor output via representational knowledge and executive functions (Keeler and Robbins 2011). At the most basic level, HIV-1 seropositive individuals (Minassian et al. 2013) display prominent alterations in preattentive processes (sensorimotor gating); deficits which have been translationally modeled across multiple biological systems used to model HAND (e.g., HIV-1 Tg rat: Moran et al. 2013, McLaurin et al. 2017, 2018; stereotaxic injections of HIV-1 viral proteins: Fitting et al. 2006a, 2006b; gp120 transgenic mice: Henry et al. 2014, Bachis et al. 2016; Tat transgenic mice: Paris et al. 2015). Alterations in the core components of cognitive function, including key components of both representational knowledge (e.g., attention, long-term episodic memory) and executive function (e.g., flexibility, inhibition), have also been reported in clinical (e.g., Heaton et al. 2011; Maki et al. 2015; Kanmogne et al. 2018) and preclinical (e.g., Lashomb et al. 2009; Moran et al. 2014; Repunte-Canonigo et al. 2014; McLaurin et al. 2018, 2019a) studies. Given the componential relationship between neurocognitive functions, examining the effect of SE on the core components of cognitive function (i.e., preattentive processes, stimulus-response learning, sustained attention, and selective attention) will provide one critical test of its therapeutic potential for HAND in the post-cART era.

In light of previous work, the goals of the present study were twofold: 1) Utilizing the HIV-1 transgenic (Tg) rat, developed by Reid et al. (2001), to assess whether SE exhibits greater therapeutic efficacy when treatment is initiated relatively early (i.e., between 2 and 3 months of age) in the course of HIV-1 viral protein exposure; 2) To determine whether the therapeutic utility of SE generalizes across multiple neurocognitive domains, including preattentive processes, stimulus-response learning, sustained attention, and selective attention. It was hypothesized that relatively early initiation of SE would ameliorate neurocognitive impairments across multiple neurocognitive domains in a subset of HIV-1 Tg animals; an effect that would enhance cognitive function to the level of controls. Understanding the generalizability of SE across ages (i.e., 2–3 months of age vs. 6–8 months of age (Moran et al. 2019)) and neurocognitive domains may aid in the development of an efficacious adjunctive therapeutic for HAND in the post-cART era.

## Methods

### Experimental Design

**Fig. 1:**
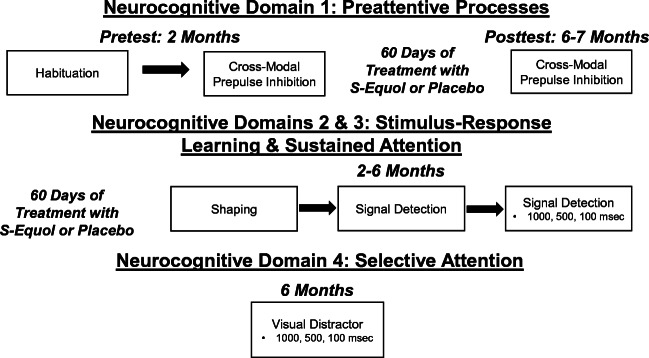
Schematic of the Experimental Design

An experimental timeline for SE treatment and neurocognitive assessments is illustrated in Fig. 1.

### Animals

At approximately two months of age, ovariectomized (OVX) female Fischer (F344/N; Harlan Laboratories Inc., Indianapolis, IN) HIV-1 Tg (*n* = 20) and control (*n* = 20) rats were delivered to the animal vivarium in two separate batches, one week apart in delivery and age. All animals were pair- or group-housed throughout the duration of experimentation. Rats were handled for one week prior to beginning neurocognitive assessments.

All HIV-1 Tg and control animals were OVX at Harlan Laboratories prior to arrival at the animal vivarium. OVX animals and a minimal phytoestrogen diet (≤20 ppm; Teklad 2020X Global Extruded Rodent Diet (Soy Protein-Free)) were utilized to preclude the potential confounding effect of endogenous hormones. Rodent food and water were available ad libitum throughout the pretest cross-modal prepulse inhibition (PPI) assessment. Animals were placed under food restriction, to maintain 85% body weight, one week prior to beginning operant testing. Rodent food was again provided ad libitum at the conclusion of operant testing and during the posttest cross-modal PPI assessment.

Guidelines established in the Guide for the Care and Use of Laboratory Animals of the National Institute of Health (NIH) were utilized for the maintenance of animals in AAALAC-accredited facilities. The animal vivarium was maintained at 21° ± 2°C, 50% ± 10% relative humidity and had a 12-h light:12-h dark cycle with lights on at 0700 h (EST). The Institutional Animal Care and Use Committee (IACUC) of the University of South Carolina approved the project protocol under federal assurance (#D16–00028).

### S-Equol

SE was obtained from Cayman Chemical Company (Ann Arbor, MI) and incorporated into 90 mg sucrose pellets by Bio-Serv (Frenchtown, NJ). Each sucrose pellet contained 0.05 mg SE. The placebo group received plain 90 mg sucrose pellets, which were also obtained from Bio-Serv.

At approximately 2–3 months of age and one week prior to beginning operant training, animals began daily treatment with SE or placebo. Animals were randomly assigned to either the SE or placebo group (Control: SE, *n* = 10, Vehicle, *n* = 10; HIV-1 Tg: SE, *n* = 10, Vehicle, *n* = 10). HIV-1 Tg and control animals treated with SE received four 90 mg sucrose pellets for a daily oral dose of 0.2 mg of SE. A dose-response experimental design previously revealed a linear dose-response with the most effective dose at 0.2 mg SE (Moran et al. 2019). Furthermore, the dose selected yielded a daily amount of 0.25–1.0 mg/kg SE; an amount equivalent to a 2.5–10 mg dose in a 60 kg human (Cf., most elderly Japanese have a daily isoflavone intake of 30–50 mg, Akaza 2012).The placebo group received four 90 mg sucrose pellets. Each rat was administered its treatment at least an hour after neurocognitive assessments and typically consumed their pellets within seconds. Animals were treated after neurocognitive assessments to promote long-term remodeling of neuronal circuitry by preventing synapse loss induced by HIV-1 viral proteins (e.g., Bertrand et al. 2014, 2015). Treatment was continued for 60 days.

### Neurocognitive Domain 1: Preattentive Processes (Prepulse Inhibition)

#### Apparatus

A 10 cm-thick double-walled, 81 × 81 × 116-cm isolation cabinet (external dimensions) (Industrial Acoustic Company, INC., Bronx, NY) enclosed the startle platform (SR-Lab Startle Reflex System, San Diego Instruments, Inc., San Diego, CA), providing over 30 dB(A) of sound attenuation relative to the external environment. The ambient sound level in the chamber, in the absence of any stimuli, was 22 dB(A). A high-frequency loudspeaker of the SR-Lab system (Radio Shack model #40-1278B) was mounted inside the isolation cabinet 30-cm above the Plexiglas test cylinder for the presentation of all discrete auditory prepulses and startling stimuli (white noise, frequency range of 5 k–16 k Hz). A microphone was placed inside the Plexiglas cylinder for the measurement and calibration of sound levels (Sound Level Meter: model #2203, Bruël & Kjaer, Norcross, GA). A 22 lx white LED light (Light meter model #840006, Sper Scientific, Ltd., Scottsdale, AZ) was affixed on the isolation cabinet wall in front of the Plexiglas test cylinder for the presentation of discrete visual prepulses. The animal’s whole body startle response to the auditory startle stimulus produced deflection of the Plexiglas test cylinder; a deflection that was converted into analog signals by a piezoelectric accelerometer integral to the bottom of the cylinder. Response signals were digitized (12 bit A to D) and saved to a hard disk. The SR-LAB Startle Calibration System was utilized to calibrate all response sensitivities.

#### Procedure

##### Habituation

At approximately 2 months of age, a 36-trial auditory startle test session was conducted to habituate animals to assessment procedures, as well as to the auditory startling stimulus. Habituation was administered beginning with a 5-min acclimation period in the dark with 70 dB(A) background white noise. Subsequently, 36 trials of a 100 dB(A) white noise stimulus (20 msec duration) were presented. The intertrial interval (ITI) was fixed at 10-s. Although HIV-1 Tg animals exhibited an overall decreased startle response relative to controls, no significant differences in the rate of habituation were observed (*p* > 0.05; data not shown).

##### Cross-Modal Prepulse Inhibition

HIV-1 Tg and control animals were assessed for PPI of the auditory startle response (ASR) using both auditory and visual prepulse stimuli at 2 months of age (prior to beginning SE treatment) and at 6–7 months of age. The assessment was conducted similar to our prior publication (Moran et al. 2013). In brief, PPI was assessed during a 30-min test session that began with a 5-min acclimation period in the dark with 70 dB(A) background white noise. After the acclimation period, six pulse-only ASR trials with a fixed 10-s ITI were presented. Seventy-two testing trials were subsequently presented, including an equal number of auditory and visual prepulse trials, arranged using an ABBA counterbalanced order of presentation. Testing trials were presented in 6-trial blocks, interdigitated using a Latin-square experimental design, with interstimulus intervals (ISI) of 0, 8, 40, 80, 120, and 4000 msec and a variable ITI (15–25 s). Control trials, including both the 0 and 4000 msec ISI trials, provided a reference ASR within the test session. Mean peak ASR amplitude values were collected for analysis.

### Neurocognitive Domains 2 & 3: Stimulus-Response Learning and Sustained Attention (Signal Detection Operant Task)

#### Apparatus

HIV-1 Tg and control animals were trained and assessed in a signal detection operant task using 22 operant boxes located inside sound-attenuating chambers (Med Associates, Inc., Fairfax, VT). The front wall of the operant chambers included a 45 mg pellet dispenser, two retractable levers, and three panel lights (22 lx). The rear wall of the operant chambers had a house light (5.5 lx). A PC and Med-PC for Windows software (V 4.1.3; Med Associates Inc., Fairfax, VT) controlled the presentation of signals, lever operation, reinforcement delivery, and data collection.

#### Procedure

##### Shaping

At approximately 2 months of age, HIV-1 Tg and control animals were trained to lever-press using a standard shaping response protocol (Moran et al. 2014). In brief, a fixed ratio 1 (FR-1) schedule of reinforcement was used to train animals to press both levers for a reinforcer (i.e., 45 mg sucrose pellet). An animal was not reinforced for more than five consecutive presses on a single lever to prevent side bias. All HIV-1 Tg and control animals successfully acquired shaping within 36 days by achieving the criterion of at least 40 reinforcers during the 42-min test session for three consecutive days, with less than 20% variance across days. Following the successful completion of the shaping response protocol, animals were promoted to the signal detection operant task.

##### Signal Detection Operant Task

The signal detection operant task, tapping sustained attention, employed three vigilance programs, initially described by McGaughy and Sarter (1995), that trained animals to discriminate between signal (i.e., central panel light illumination) and non-signal (i.e., no illumination) trials. Methodology utilized in the present study is similar to our prior publication (McLaurin et al. 2019a) with minor modifications.

In brief, each signal detection operant session began with a 5-min acclimation period in the dark. The presentation of signals (central panel light illumination) and non-signals (no illumination) was randomized across trials throughout the session, with varying ITIs (9 ± 3 s), during which time the levers remained retracted. Two seconds after each trial began, levers were extended until the animal made a response, or 6 s elapsed, whichever occurred first. For half of the animals, lever presses on the left lever during signal trials (Hits) and on the right lever during non-signal trials (Correct Rejections) were rewarded with a 45 mg sucrose pellet. In the same manner, responses on the right lever during signal trials (Misses) and on the left lever during non-signal trials (False Alarms) were not reinforced. The other half of the animals were trained using the reverse set of contingencies.

During the first vigilance program, consisting of 160 trials per session, termination of the central panel light illumination (i.e., the signal) was contingent upon a response. In the second vigilance program, also consisting of 160 trials per session, the central panel light was illuminated for 1 s. Correction trials and force-choice trials, which occurred after an incorrect response, were an integral component of the first two vigilance programs. Specifically, during correction trials, an animal was provided with up to three repetitions of the trial. If an animal failed to respond appropriately to the correction trials, a forced-choice trial occurred during which the same stimulus type (i.e., signal or non-signal) was repeated, but only the correct lever was extended. The lever remained extended until a response was made or 2 min elapsed, whichever occurred first. The third vigilance program consisted of 162 trials per session and manipulated the signal duration (i.e., 1000, 500, 100 msec) across trials using a block randomized experimental design. Correction trials and forced-choice trials were removed in the third vigilance program.

HIV-1 Tg and control animals were trained on each vigilance program, assessing signal detection, until achieving at least 70% accuracy on three consecutive test sessions or until 76 days. Accuracy was calculated as the total number of hits and correct rejections divided by the total number of correct and incorrect responses in a session.

### Neurocognitive Domain 4: Selective Attention (Visual Distractor Task)

#### Apparatus

The assessment of selective attention in HIV-1 Tg and control animals was conducted in the operant chambers described above.

#### Procedure

For the assessment of selective attention, the signal detection operant task with varying signal durations was divided into three trial blocks, each containing 54 trials. During the second trial block, a 1.5 s visual distractor stimulus was presented at the beginning of each trial. The visual distractor stimulus onset and offset was 1 s prior to and 0.5 s after the signal onset (center panel light), for a 1.5 s total duration. The house light was used as the visual distractor, with an intensity of 5.5 lx measured from the center of the chamber at the level of the animal’s height. The assessment was conducted for three consecutive sessions.

### Statistical Analysis

Statistical techniques, including analysis of variance (ANOVA) and regression, were utilized for the analysis of all data (SAS/STAT Software 9.4, SAS Institute, Inc., Cary, NC; SPSS Statistics 26, IBM Corp., Somer NY; GraphPad Software, Inc., La Jolla, CA). Figures were created using GraphPad Prism 5 (GraphPad Software, Inc., La Jolla, CA). For all statistical analyses, significance was set at an alpha criterion of *p* ≤ 0.05. Orthogonal decompositions or the Greenhouse-Geisser *df* correction factor (Greenhouse and Geisser 1959) were used for variables that classically violate compound symmetry assumptions.

A mixed-factor ANOVA was conducted to examine the effect of the HIV-1 transgene and/or SE treatment on body weight during food restriction (SPSS Statistics 26, IBM Corp., Somer NY). Genotype (HIV-1 Tg vs. control) and treatment (SE vs. placebo) were included as between-subject’s factors, while age served as a within-subject’s factor. Furthermore, linear regression analyses, fit with 95% confidence intervals (CI), were also utilized to directly assess functional form (GraphPad Software, Inc., La Jolla, CA).

Two approaches were utilized to assess the therapeutic efficacy of SE for HAND. First, statistical analyses were conducted on all animals (Control: SE: *n* = 10; Placebo: *n* = 10; HIV-1 Tg: SE: *n* = 10; Placebo: *n* = 10). Second, when statistically significant effects were not observed in the overall analysis, complementary analyses including a subset of animals were conducted. Based on previous work (Moran et al. 2019), our a priori hypothesis was that SE treatment would only mitigate neurocognitive deficits in a subset of HIV-1 Tg animals. Furthermore, a priori planned comparisons were conducted to evaluate the genotype deficit (i.e., Control Placebo vs. HIV-1 Tg Placebo) and the effect of SE (i.e., amelioration of neurocognitive deficits to approximate control animals treated with placebo; Control Placebo vs. HIV-1 Tg SE).

The temporal process of acquisition (i.e., the number of days to meet criterion), an assessment of stimulus-response learning, was analyzed using a generalized linear mixed effects model with a Poisson distribution (SAS/STAT Software 9.4, SAS Institute, Inc., Cary, NC). Linear regression analyses, fit with 95% CI, were also utilized to directly assess functional form (GraphPad Software, Inc., La Jolla, CA). Genotype (HIV-1 Tg vs. control) and treatment (SE vs. placebo) were included as between-subject’s factors.

A mixed-factor ANOVA with a compound symmetry covariance structure (SAS/STAT Software 9.4, SAS Institute, Inc., Cary, NC) was utilized for the assessment of preattentive processes and sustained attention. Selective attention was assessed by conducting a mixed-factor ANOVA in SPSS (SPSS Statistics 26, IBM Corp., Somer NY). Genotype (HIV-1 Tg vs. control) and treatment (SE vs. placebo) served as between-subject’s factors. ISI (0, 8, 40, 80, 120, 4000), response type (hits, misses), signal duration (1000, 500, 100 msec), and time (Pretest Assessment vs. Posttest Assessment) served as the within-subject’s factors, as appropriate. ISI, trial, signal duration, and time were included as random effects in the analysis, as appropriate. For the analysis of preattentive processes, the dependent variable (i.e., mean peak ASR amplitude) was log transformed. For the analysis of sustained attention, the relative frequency of hits and misses served as the dependent variable. Furthermore, for the analysis of selective attention, the relative frequency of hits and misses during the second trial block (i.e., when the distractor was present), served as the dependent variable.

## Results

### Body Weight: Somatic Growth

**Fig. 2:**
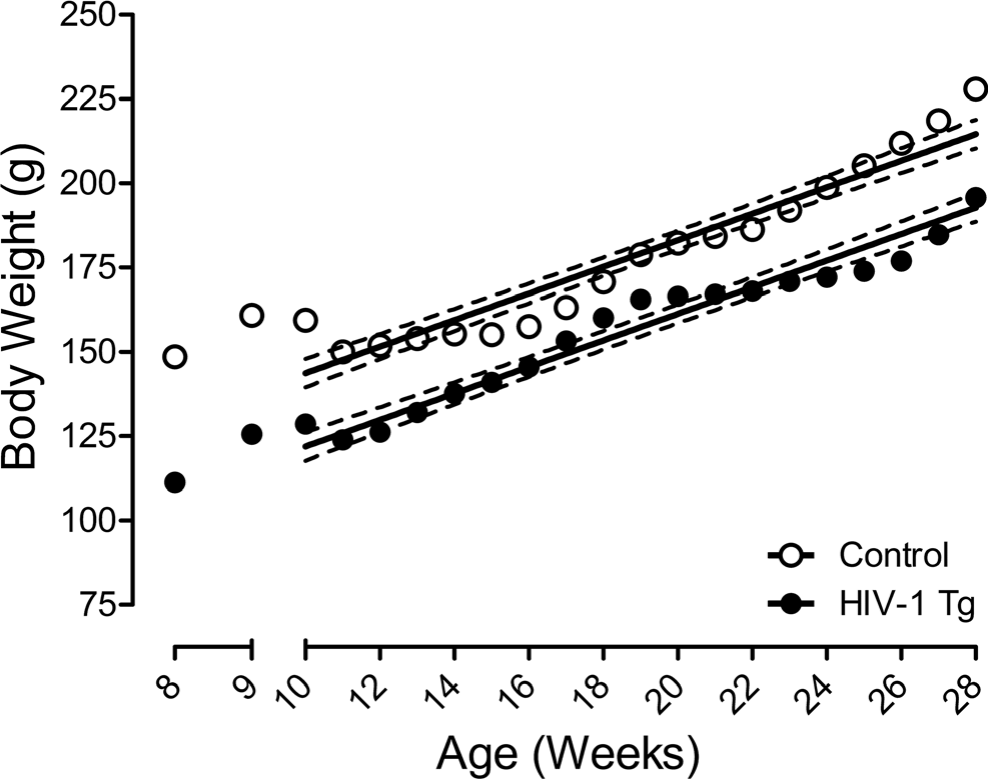
Mean body weight is illustrated as a function of genotype (HIV-1 Tg vs. Control) and age (±95% CI). HIV-1 Tg animals weighed significantly less than the control group across the testing period. Both groups increased significantly in body weight across this period and did not differ in their rates of growth. The x-axis break at 65 days indicates the point at which animals began food restriction, prior to testing

Body weight was assessed as a measurement of somatic growth in HIV-1 Tg and control animals from 8 weeks of age through 28 weeks of age (Fig. 2). At approximately 10 weeks of age, animals were placed on food restriction prior to beginning the signal detection operant task. During the implementation of food restriction (10 Weeks to 28 Weeks of Age), HIV-1 Tg animals, independent of treatment (i.e., SE vs. placebo) weighed significantly less than control animals (Main Effect of Genotype: *F*(1,36) = 56.6, *p* ≤ 0.001). Neither a significant main effect of treatment (*p* > 0.05) nor a genotype x treatment interaction (*p* > 0.05) were observed.

Both HIV-1 Tg and control animals exhibited steady growth across development, evidenced by a linear increase in body weight (Control: *R*^2^ = 0.92; HIV-1 Tg: *R*^2^ = 0.95). Most notably, however, no significant differences between HIV-1 Tg and control animals were observed in the rate of somatic growth (i.e., β_1_; *F*(1,34) = 2.4, *p* > 0.05). Thus, although HIV-1 Tg animals weighed significantly less than their control counterparts, no significant alterations in the growth trajectory of HIV-1 Tg animals were observed. Furthermore, independent of genotype, treatment with SE did not alter somatic growth.

### Neurocognitive Domain 1: Preattentive Processes

**Fig. 3:**
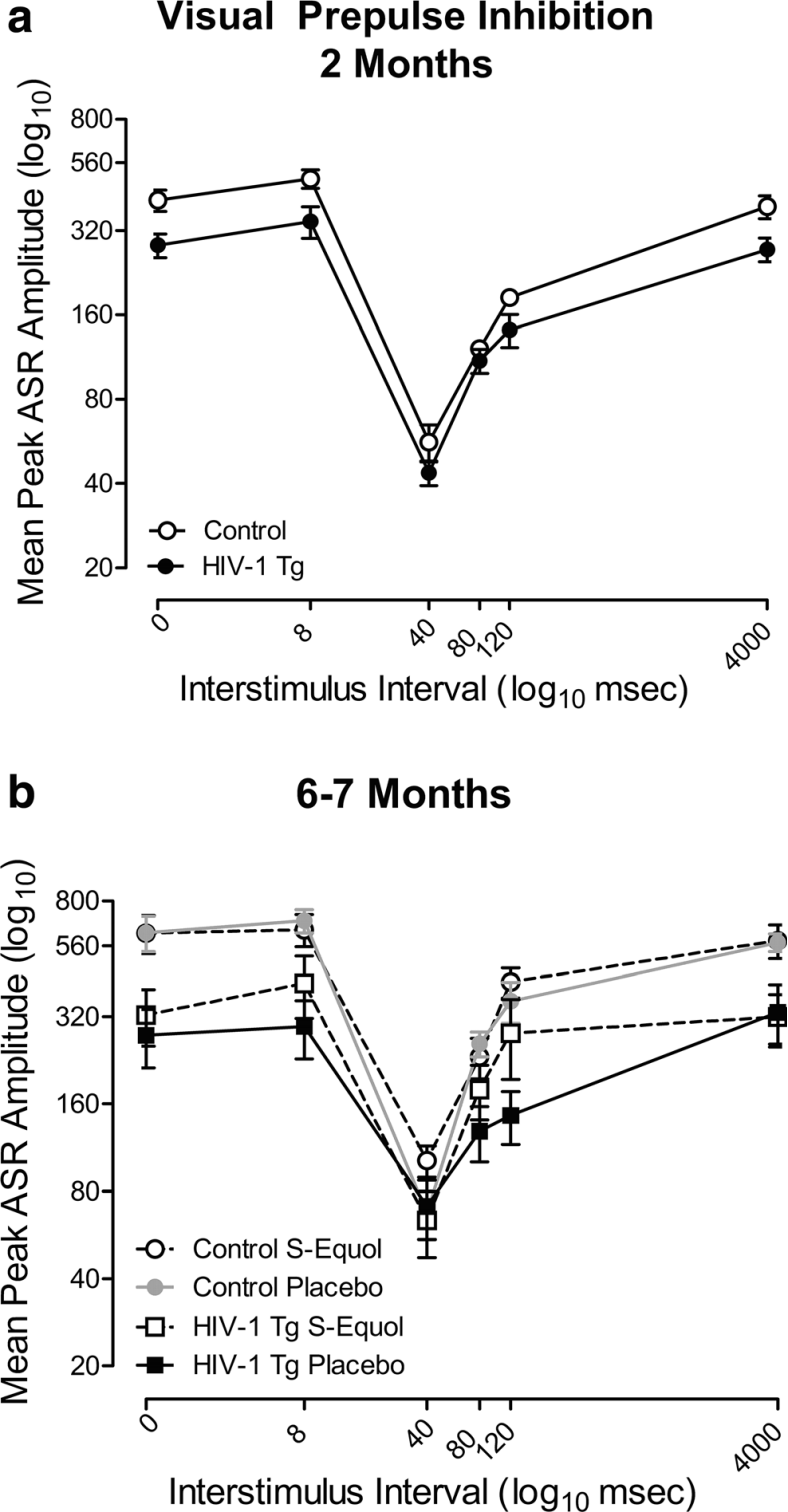
Mean peak ASR amplitude for prepulse inhibition (PPI), an assessment of preattentive processes, with a visual prepulse is illustrated as a function of genotype (HIV-1 Tg vs. Control), age, and treatment (S-Equol (SE) vs. placebo; ±SEM). At 2 months of age **(a)**, no significant alterations in PPI were observed. However, at 6–7 months of age **(b)**, HIV-1 Tg animals treated with placebo exhibited a prominent deficit in temporal processing relative to control animals; a deficit which was ameliorated in HIV-1 Tg animals treated with SE

HIV-1 Tg and control animals were assessed in PPI, tapping preattentive processes, prior to SE treatment (i.e., Pretest Assessment, 2 months of age; Fig. 3a) and after SE treatment (i.e., Posttest Assessment, 6–7 months of age; Fig. 3b). At both the pretest and posttest assessments, all animals, independent of genotype and/or treatment, displayed maximal inhibition at the 40 msec ISI. Observations of robust inhibition to the visual prepulse at the 40 msec ISI during both test sessions supports the integrity of visual system function. However, significant alterations in the development of perceptual sharpening were observed in HIV-1 Tg animals treated with placebo; deficits that were restored with SE treatment.

Control animals, independent of treatment, exhibited age-related perceptual sharpening evidenced by a sharper ISI function at the posttest assessment relative to the pretest assessment. In sharp contrast, HIV-1 Tg animals treated with placebo failed to develop perceptual sharpening. Specifically, at the posttest assessment, HIV-1 Tg animals treated with placebo displayed a relative insensitivity to the manipulation of ISI, evidenced by a flattening of the ISI function relative to control animals. Treatment with SE, however, ameliorated alterations in the development of perceptual sharpening in the population of HIV-1 Tg animals sampled.

The overall mixed-model ANOVA conducted on mean peak response amplitude (Control: SE: *n* = 10; Placebo: *n* = 10; HIV-1 Tg: SE: *n* = 10; Placebo: *n* = 10) confirmed our observations, revealing a statistically significant genotype x treatment x ISI interaction [*F*(5,180) = 3.2, *p* ≤ 0.008]; an interaction which was subsequently examined by conducting a priori planned comparisons (i.e., Control Placebo vs. HIV-1 Tg Placebo, Control Placebo vs. HIV-1 Tg SE). Comparison of HIV-1 Tg and control animals treated with placebo revealed a statistically significant genotype x ISI interaction [*F*(5,90) = 5.5, *p* ≤ 0.001]; an interaction not observed when HIV-1 Tg treated with SE were compared with control animals treated with placebo [*p* > 0.05]. Thus, treatment with SE ameliorated the marked impairment in the development of perceptual sharpening in the population of HIV-1 Tg animals sampled.

### Neurocognitive Domains 2 & 3: Stimulus-Response Learning and Sustained Attention

#### Stimulus-Response Learning: Temporal Process of Acquisition

**Fig. 4:**
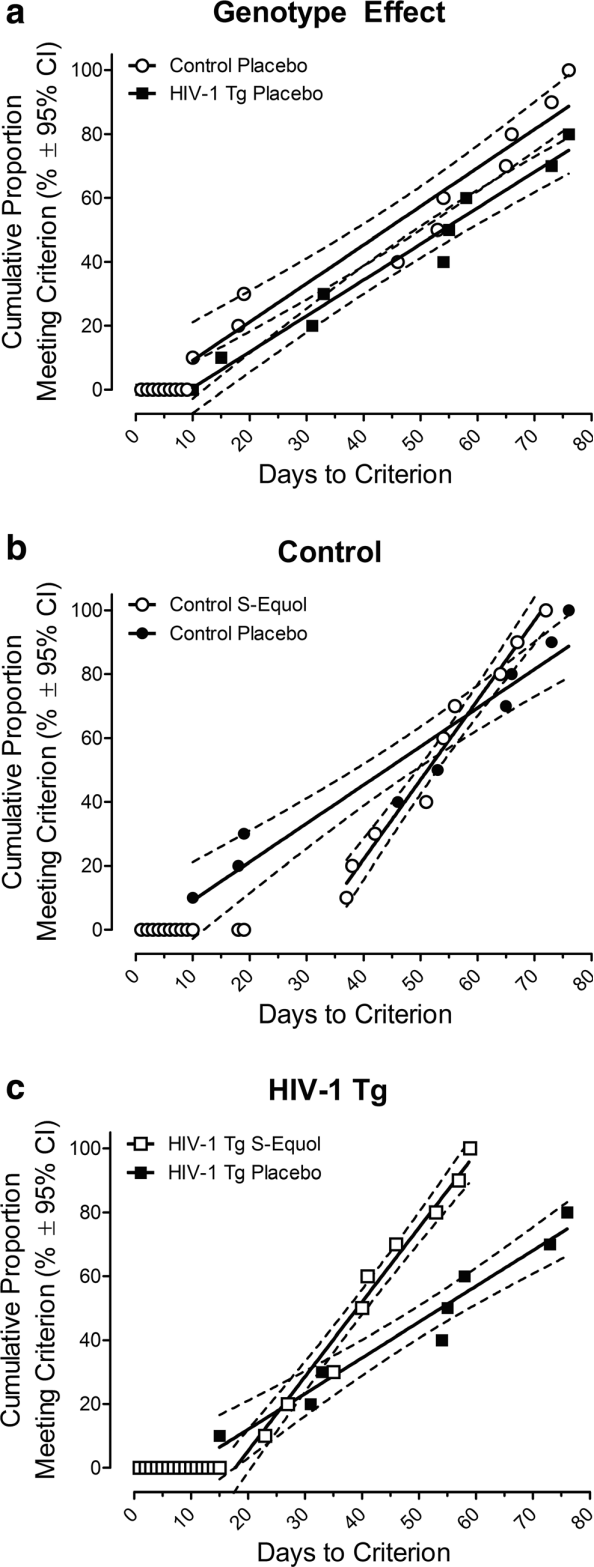
The number of days required to meet criterion in the signal detection operant task is illustrated as a function of genotype (HIV-1 Tg vs. Control) and treatment (S-Equol (SE) vs. placebo; ±95% Confidence Intervals). **(a)** HIV-1 Tg animals treated with placebo displayed a pronounced deficit in stimulus-response learning, acquiring the task significantly slower than their control counterparts. **(b)** In control animals, no significant treatment differences were observed in the temporal process of acquisition. **(c)** In HIV-1 Tg animals, treatment with SE significantly enhanced the temporal process of acquisition, evidenced by faster acquisition relative to HIV-1 Tg animals treated with placebo

Animals were required to meet criterion of 70% accuracy for three consecutive test sessions to successfully acquire each program in the signal detection operant task. The task included a series of three vigilance programs. Independent of treatment, control animals (SE: *n* = 10; Placebo: *n* = 10) acquired the signal detection task within 76 test sessions. Furthermore, HIV-1 Tg animals treated with SE successfully acquired the signal detection task within 59 test sessions. In sharp contrast, only 80% (*n* = 8) of the HIV-1 Tg animals treated with placebo (*n* = 10) were able to successfully acquire the task. The temporal process of acquisition for all groups, independent of genotype and/or treatment, was well-described by a first-order polynomial (*R*^2^s > 0.93; Fig. 4).

Significant genotype differences were observed between HIV-1 Tg and control animals treated with placebo (Fig. 4A). Specifically, HIV-1 Tg animals acquired the signal detection task significantly slower than their control counterparts, evidenced by significant differences in the parameters of the function [*F*(2,15) = 6.1, *p* ≤ 0.01]. HIV-1 Tg animals, therefore, exhibited a marked impairment in stimulus-response learning relative to control animals.

Treatment with SE, however, ameliorated deficits in stimulus-response learning in all HIV-1 Tg animals. Specifically, a generalized linear mixed model with a Poisson distribution (Control: SE: *n* = 10; Placebo: *n* = 10; HIV-1 Tg: SE: *n* = 10; Placebo: *n* = 8) revealed a significant genotype x treatment interaction [*F*(1,34) = 10.5, *p* ≤ 0.003]; an interaction that was further examined via complementary analyses of each genotype. In control animals (Fig. 4B), no significant treatment differences were observed in the temporal process of acquisition [*p* > 0.05]. In sharp contrast, in HIV-1 Tg animals, treatment with SE significantly enhanced the temporal process of acquisition (Fig. 4C). Specifically, HIV-1 Tg animals treated with SE acquired the signal detection task significantly faster than HIV-1 Tg animals treated with placebo, evidenced by significant differences in the parameters of the first-order polynomial [*F*(2,14) = 59.1, *p* ≤ 0.001] and a main effect of treatment [*F*(1,16) = 7.4, *p* ≤ 0.015]. Thus, treatment with SE ameliorated the marked impairment in stimulus-response learning in HIV-1 Tg animals.

#### Sustained Attention: Signal Detection

The effect of the HIV-1 transgene and/or SE treatment was examined by averaging each animal’s performance in the signal detection operant task with varying signal durations (i.e., 1000, 500, 100 msec) across the final three consecutive sessions. To directly assess the temporal components of attention, hits and misses (i.e., response types occurring during signal trials) were the focus of statistical analyses.

The overall mixed-model ANOVA, conducted on the relative frequency of hits and misses at each signal duration, in all animals (Control: SE: *n* = 10; Placebo: *n* = 10; HIV-1 Tg: SE: *n* = 10; Placebo: *n* = 10) failed to reveal a statistically significant main effect of treatment, genotype x treatment interaction, or higher-order interactions with genotype and treatment (*p* > 0.05).

Given the a priori hypothesis that SE treatment may only mitigate sustained attention deficits in a subset of HIV-1 Tg animals, additional analyses were conducted. Specifically, analyses were conducted on all control animals (SE: *n* = 10; Placebo: *n* = 10), all HIV-1 Tg animals treated with placebo (*n* = 10), and the top performing 50% of HIV-1 Tg animals treated with SE (*n* = 5). Performance in HIV-1 Tg animals treated with SE was determined by calculating the area of the signal detection curve where the number of hits was greater than the number of misses (McLaurin et al. 2019b).

**Fig. 5:**
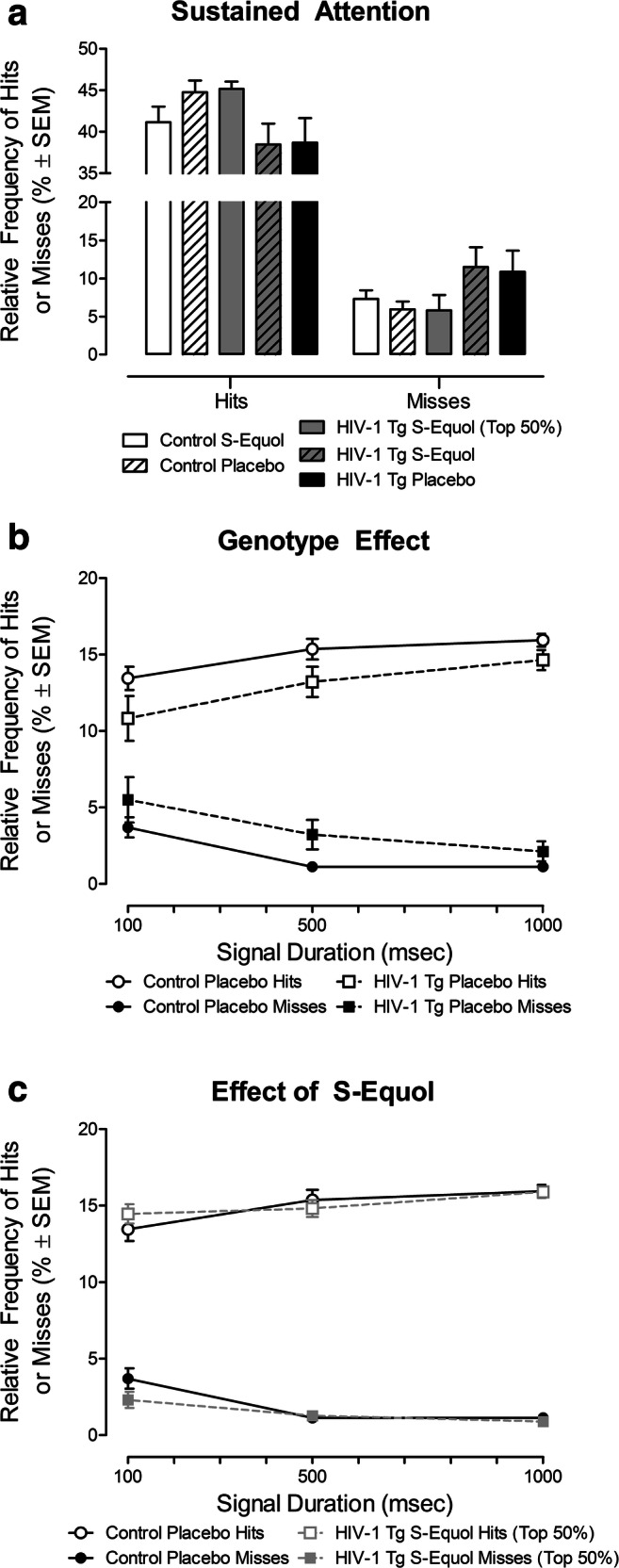
Temporal components of sustained attention were assessed by evaluating hits and misses (i.e., correct and incorrect responses, respectively, during signal trials) **(a)** The number of hits and misses during the final 3 days in the signal detection operant task are presented independent of signal duration using relative frequencies (mean ± SEM) as a function of genotype (HIV-1 Tg vs. Control) and treatment (S-Equol (SE) vs. Placebo). **(b)** HIV-1 animals treated with placebo exhibited a prominent deficit in sustained attention, evidenced by a decreased relative frequency of hits and an increased relative frequency of misses, independent of signal duration, relative to control animals treated with placebo. **(c)** Treatment with SE, however, ameliorated sustained attention deficits in a subset (i.e., 50%) of HIV-1 Tg animals. Independent of signal duration, HIV-1 Tg animals treated with SE exhibited frequencies of hits and misses that approximated control animals treated with placebo

SE treatment between 2 and 3 months of age significantly enhanced sustained attention in 50% of the HIV-1 Tg animals (Fig. 5a), evidenced by a statistically significant genotype x treatment x response type interaction [*F*(1,31) = 16.7, *p* ≤ 0.001]. Complementary analyses were conducted to determine the locus of the interaction by 1) conducting a priori planned comparisons (i.e., Control Placebo vs. HIV-1 Tg Placebo, Control Placebo vs. HIV-1 Tg SE) and 2) examining each response type (i.e., Hits, Misses). First, comparison of HIV-1 Tg and control animals treated with placebo (Fig. 5b) revealed a statistically significant genotype x response type interaction [*F*(1,18) = 11.44, *p* ≤ 0.003]; an interaction not observed when HIV-1 Tg animals treated with SE were compared with control animals treated with placebo (Fig. 5c [*p* > 0.05]). Second, each response type (i.e., Hits, Misses) was examined individually, revealing a genotype x treatment interaction for both hits [*F*(1,31) = 4.9, *p* ≤ 0.04] and misses [*F*(1,31) = 4.1, *p* ≤ 0.05]. Independent of signal duration (i.e., mean shift), treatment with SE increased the relative frequency of hits and decreased the relative frequency of misses in HIV-1 Tg animals. Thus, in HIV-1 Tg animals, SE treatment ameliorated sustained attention deficits by improving attention to the stimulus (i.e., hits) and preventing lapses in attention (i.e., misses); an effect that was observed a month after the cessation of treatment.

### Neurocognitive Domain 4: Selective Attention

Selective attention was assessed for three consecutive days by presenting a visual distractor at the beginning of each trial in the second trial block (i.e., Trials 54–108). The effect of the HIV-1 transgene and/or SE treatment were examined by averaging each animal’s performance across all three assessments. To directly assess the temporal components of attention, hits and misses (i.e., response types occurring during signal trials) were the focus of statistical analyses. Task validation, however, utilized false alarms, a response type occurring during non-signal trials.

**Fig. 6:**
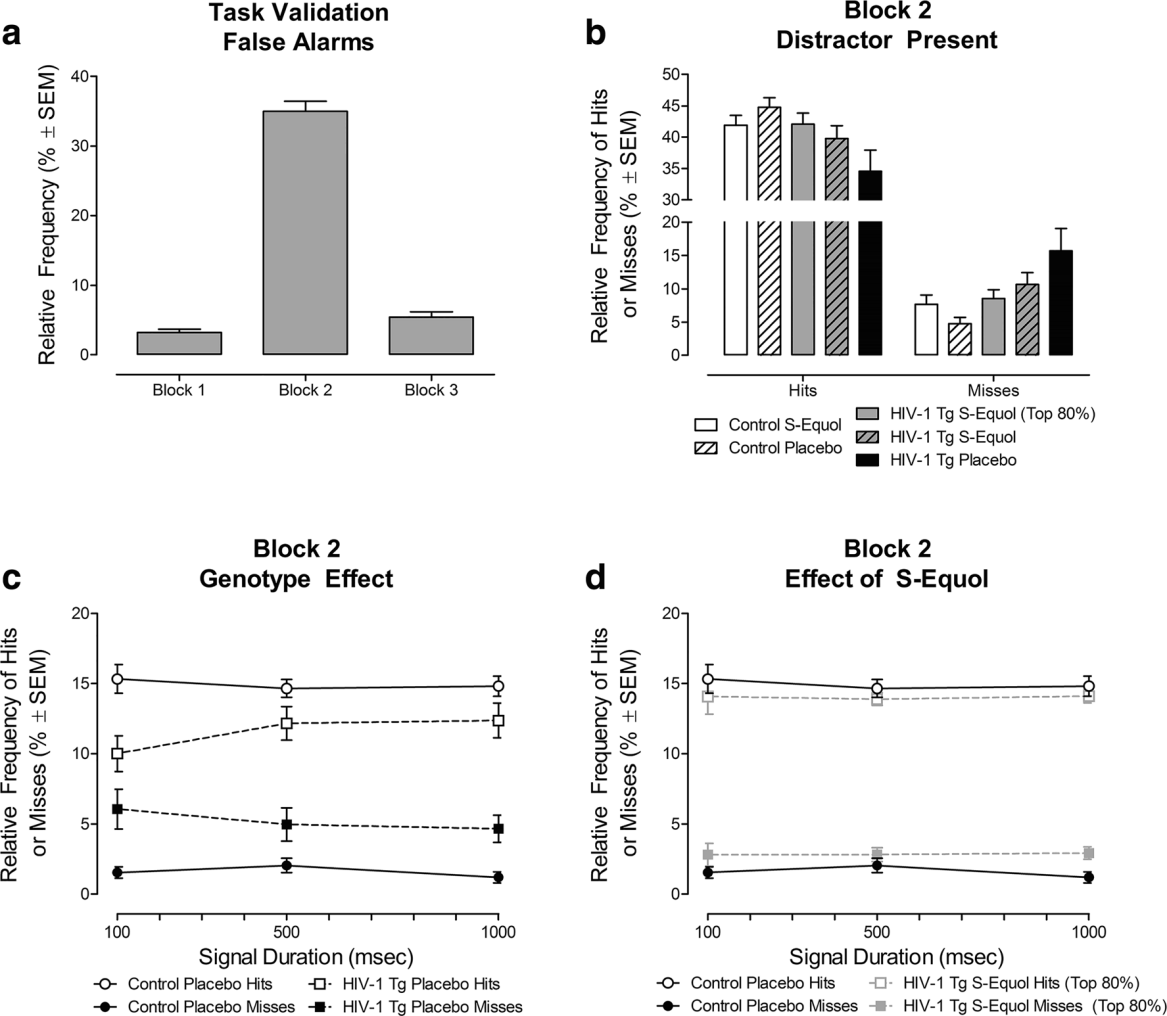
**(a)** The number of false alarms, collapsed across genotype and treatment, is illustrated across 3 trial blocks in the signal detection task. During Block 2, a visual distractor was presented for the assessment of selective attention. Animals exhibited a significantly greater number of false alarms during Block 2 relative to Block 1 or Block 3 validating the assessment of selective attention. **(b)** The number of hits and misses during the visual distractor task are presented during Block 2, independent of signal duration, using relative frequencies (mean ± SEM) as a function of genotype (HIV-1 Tg vs. Control) and treatment (S-Equol (SE) vs. Placebo). **(c)** HIV-1 animals treated with placebo exhibited a prominent deficit in selective attention, evidenced by a decreased relative frequency of hits and an increased relative frequency of misses during the second trial block, independent of signal duration, relative to control animals treated with placebo. **(d)** Treatment with SE, however, ameliorated selective attention deficits in a subset (i.e., 80%) of HIV-1 Tg animals. Independent of signal duration, HIV-1 Tg animals treated with SE exhibited frequencies of hits and misses that approximated control animals treated with placebo

The number of false alarms during each trial block were examined to validate the assessment of selective attention in all animals (Control: SE: *n* = 10; Placebo: *n* = 10; HIV-1 Tg: SE: *n* = 10; Placebo: *n* = 10; Fig. 6a). A significant main effect of trial block [*F*(2,72) = 382.2, *p*_GG_ ≤ 0.001] with a prominent quadratic component [*F*(1,36) = 458.8, *p* ≤ 0.001] supports the assessment of selective attention. During the second trial block (i.e., when the distractor was present), a dramatic increase in the relative frequency of false alarms was observed relative to either the first or third trial blocks.

The overall mixed-model ANOVA, conducted on the relative frequency of hits and misses during Block 2, in all animals (Control: SE: *n* = 10; Placebo: *n* = 10; HIV-1 Tg: SE: *n* = 10; Placebo: *n* = 10) failed to reveal a statistically significant main effect of treatment, genotype x treatment interaction, or higher-order interactions with genotype and treatment (*p* > 0.05).

Given the a priori hypothesis that SE treatment may only mitigate selective attention deficits in a subset of HIV-1 Tg animals, additional analyses were conducted. Specifically, analyses were conducted on all control animals (SE: *n* = 10; Placebo: *n* = 10), all HIV-1 Tg animals treated with placebo (*n* = 10), and the top performing 80% of HIV-1 Tg animals treated with SE (*n* = 8). Performance in HIV-1 Tg animals treated with SE was determined by calculating the area of the signal detection curve where the number of hits was greater than the number of misses (McLaurin et al. 2019b).

SE treatment between 2 and 3 months of age significantly enhanced selective attention in 80% of the HIV-1 Tg animals (Fig. 6b), evidenced by a statistically significant genotype x treatment x response type interaction [*F*(1,34) = 5.7, *p*_GG_ ≤ 0.023]. Complementary analyses were conducted to determine the locus of the interaction by 1) conducting a priori planned comparisons (i.e., Control Placebo vs. HIV-1 Tg Placebo, Control Placebo vs. HIV-1 Tg SE) and 2) examining each response type (i.e., Hits, Misses). First, comparison of HIV-1 Tg and control animals treated with placebo (Fig. 6c) revealed a statistically significant genotype x response type interaction [*F*(1,18) = 8.8, *p*_GG_ ≤ 0.008]; an interaction not observed when HIV-1 Tg animals treated with SE were compared with control animals treated with placebo (Fig. 6d [*p* > 0.05]). Second, each response type (i.e., Hits, Misses) was examined individually, revealing a statistically significant genotype x treatment interaction for both hits [*F*(1,34) = 5.3, *p* ≤ 0.028] and misses [*F*(1,34) = 5.8, *p* ≤ 0.021]. Independent of signal duration (i.e., mean shift), treatment with SE increased the relative frequency of hits and decreased the relative frequency of misses in HIV-1 Tg animals. Thus, in HIV-1 Tg animals, SE treatment ameliorated deficits in selective attention by improving attention to the stimulus (i.e., hits) and preventing lapses in attention (i.e., misses); an effect that was observed nearly two months after the cessation of treatment.

## Discussion

Two key questions were addressed in the HIV-1 Tg rat to critically test the therapeutic potential of the selective estrogen receptor β agonist (SERBA) SE. First, the therapeutic efficacy of SE was greater when treatment was initiated relatively early (i.e., between 2 and 3 months of age) in the course of viral protein exposure relative to later treatment initiation (i.e., between 6 and 8 months of age; Moran et al. 2019). Second, the therapeutic utility of SE generalizes across multiple neurocognitive domains, including preattentive processes, stimulus-response learning, sustained attention, and selective attention. Dependent upon neurocognitive domain, between 50% (i.e., sustained attention; 80%, selective attention) to 100% (i.e., preattentive processes, stimulus response learning) of HIV-1 Tg animals treated with SE exhibited enhanced neurocognitive function, approximating controls. Results support, therefore, the therapeutic efficacy of selectively targeting ERβ via SE treatment as an adjunctive therapeutic for HAND in the post-cART era. Critically testing novel adjunctive therapeutics is vital to understanding their advantages and opportunities for improving the treatment and diagnosis of HAND.

Neurocognitive functions, including preattentive processes, stimulus-response learning, and attention, are generally componential (Keeler and Robbins 2011). The componential relationship between neurocognitive functions has been examined in the HIV-1 Tg rat via longitudinal mediation; a study that supported preattentive processes as one of the neurobehavioral mechanisms underlying alterations in stimulus-response learning and sustained attention in the HIV-1 Tg rat (McLaurin et al. 2019b). In the present study, treatment with SE enhanced preattentive processes, to the level of controls, in the population (i.e., 100%) of HIV-1 Tg animals sampled. Enhancing lower-order cognitive processes, including preattentive processes may be more straightforward than restoring deficits in more complex cognitive processes (e.g., sustained attention, selective attention); deficits which may require treatment earlier in the course of HIV-1 viral protein exposure (e.g., PD 28) or a longer duration of treatment. Results suggest, therefore, that SE may target one of the neurobehavioral mechanisms underlying HAND, however, future studies utilizing mediation by design are critical to test this hypothesis.

A signal detection operant task, developed by McGaughy and Sarter (1995), was utilized to assess sustained and selective attention. Sustained attention, characterized by the detection of rare, unpredictable, and weak stimuli over long periods of time (Sarter et al. 2001), was examined by requiring animals to attend to a randomly presented stimulus (i.e., central panel illumination), the presence or absence of which indicated which response to make (i.e., which lever to press) to receive a reinforcer (i.e., sucrose pellet). The parametric manipulation of cue characteristics (i.e., presenting a visual distractor during the second trial block) allowed for the assessment of selective attention (Bushnell and Strupp 2009), which requires animals to process the most relevant information, while excluding or inhibiting, irrelevant information (Fuster 2008). The parametric manipulation of signal duration in both the sustained attention task and the selective attention task afforded a critical opportunity to evaluate the temporal aspects of attention. On each trial, independent of task, an animal could emit one of four response choices (i.e., hit, miss, correct rejection, or false alarm), indicative of an animal’s ability to attend to the stimulus (hit, correct rejection), a lapse of attention (miss) or a failure of response inhibition (false alarm).

Treatment with SE enhanced both sustained and selective attention, to the level of controls, in 50% and 80% of HIV-1 Tg animals, respectively, by increasing an animals’ ability to attend to the stimulus (i.e., hits) and decreasing lapses in attention (i.e., misses). In both sustained and selective attention, a subset (i.e., 50% and 80%, respectively) of HIV-1 Tg animals treated with SE exhibited a decreased relative frequency of misses and an increased relative frequency of hits; effects that were independent of signal duration. When HIV-1 Tg animals were treated with SE at a more advanced age (i.e., 6 to 8 months), the enhancement in sustained attention occurred in a smaller subset (i.e., 40%) of animals and was localized to misses at the shortest signal duration (i.e., 100 msec; Moran et al. 2019). Notably, the assessment of two attentional domains in the present study tapped multiple regions of the prefrontal cortex (PFC), with the medial PFC (mPFC) having a primary role in sustained attention (Kim et al. 2016), whereas the lateral PFC plays a more prominent role in selective attention (Kam et al. 2018). SE treatment relatively early in the course of viral protein exposure (i.e., 2 to 3 months), therefore, enhances sustained and selective attention in a global manner, targeting both hits and misses across signal durations.

The perception of time, which has been implicated as an elemental dimension of HAND (e.g., Chao et al. 2004; Matas et al. 2010; Moran et al. 2013; McLaurin et al. 2019b), is a cognitive capacity that is characterized by an organism’s sensitivity to the passage of time (Meck and Benson 2002). Utilization of a series of neurocognitive assessments (i.e., PPI, stimulus-response learning, signal detection, visual distractor task) and time interval manipulations (i.e., from milliseconds to days), as in the present study, provided a critical opportunity to evaluate the generalizability of temporal processing deficits in the HIV-1 Tg rat. Independent of neurocognitive domain, HIV-1 Tg rats treated with placebo exhibited a fundamental deficit in the perception of time relative to control animals, extending previously reported observations (e.g., Moran et al. 2013; McLaurin et al. 2016; McLaurin et al. 2019a; Moran et al. 2019). Treatment with SE, however, ameliorated alterations in the perception of time, evidenced by improvements in preattentive processes, stimulus-response learning, sustained attention and selective attention in 50% to 100% of HIV-1 Tg animals. Most notably, all of the HIV-1 Tg animals treated with SE exhibited an enhancement in preattentive processes and stimulus-response learning. While the improvement in preattentive processes likely reflects the utility of SE to target one of the neurobehavioral mechanisms underlying HAND (McLaurin et al. 2019b), the enhancement of stimulus-response learning may result from the presence of longer temporal intervals (i.e., days relative to msec).

The frontal-subcortical circuit, which includes five parallel segregated circuits linking the basal ganglia and prefrontal cortex (PFC; Alexander et al. 1986; Alexander and Crutcher 1990; Alexander 1994), has been implicated as a potential neuroanatomical substrate for temporal processing (for review, Matell and Meck 2000; Meck and Benson 2002). Specifically, excitatory glutamatergic projections from the prefrontal cortex (PFC) innervate the striatum, which receives dopaminergic projections from the ventral tegmental area and substantia nigra pars compacta (SNpc). The striatum sends GABAergic projections to the globus pallidus, which are subsequently relayed to the thalamus. Excitatory glutamatergic projections from the thalamus then innervate the PFC. Notably, the neuroanatomical regions involved in temporal processing are also involved in the neurocognitive domains assessed in the present study (e.g., preattentive processes: Ellenbroek et al. 1996; stimulus-response learning: Rolls 2004; sustained attention: Kim et al. 2016; selective attention: Kam et al. 2018). Critically, damage to frontal-subcortical circuitry has been observed in HIV-1 seropositive individuals, evidenced by impairments in executive function (e.g., Cysique et al. 2004; Heaton et al. 2011) and neurobehavioral alterations (e.g., apathy: Cole et al. 2007; Kamat et al. 2012; depression: Ciesla and Roberts 2001); results which were translationally modeled in the HIV-1 Tg rat (e.g., executive function impairments: Vigorito et al. 2007; Moran et al. 2014; McLaurin et al. 2019a; motivational dysregulation: Bertrand et al. 2018).

Mechanistically, SE may remodel the frontal-subcortical circuit at the synaptic level by targeting dendritic spines. Dendritic spines, which reflect functionality and capacity for structural change (Lai and Ip 2013), serve as the main postsynaptic compartment of excitatory synapses (Spiga et al. 2014). Long-term modifications (i.e., density, morphology) in dendritic spines may lead to the remodeling of neuronal circuitry and changes in synaptic function targeting a potential neural mechanism (i.e., synaptic dysfunction) underlying HAND (e.g., Gelman and Nguyen 2010; Gelman et al. 2012; Roscoe et al. 2014; Sinharay et al. 2017). Broadly, strong evidence suggests that 17β-estradiol remodels neural circuits by increasing dendritic spine density (e.g., Khan et al. 2013; Hao et al. 2006; Tuscher et al. 2016) and altering dendritic spine morphology (Hao et al. 2006). Specifically, 17β-estradiol promotes the growth and stability of new dendritic spines via the ERβ pathway (Wang et al. 2018) and enhances excitatory glutamatergic synapse formation (Khan et al. 2013).

In vitro studies further support the utility of phytoestrogens, including DAI, LQ, and SE to remodel neural circuits by preventing synapse loss induced by the HIV-1 viral protein, Tat (Bertrand et al. 2014, 2015). Phytoestrogens are one of the few compounds that show potential to restore synaptic connectivity after exposure to HIV-1 Tat (Bertrand et al. 2015) and do so via an ERβ specific mechanism; although other synthetic SERBA compounds have been shown to enhance recovery of neurons following damage (D’Errico et al. 2018). Furthermore, the clinical importance of SERBAs is demonstrated by their progression into clinical trials and experimental studies of other neurodegenerative diseases, including Parkinsons disease (McFarland et al. 2013) and Alzheimer’s disease (Zhao et al. 2013). The development of a therapeutic approach targeting synaptic dysfunction, therefore, may have long-term effects that lead to the remodeling of neural circuitry and enhanced cognitive function.

In conclusion, the present study critically tested the utility of SE as a novel adjunctive therapeutic for the treatment of neurocognitive impairments in HIV-1. When initiated relatively early (i.e., 2 to 3 months of age) in the course of HIV-1 viral protein exposure, SE enhances neurocognitive function in multiple neurocognitive domains. Therefore, selectively targeting ERβ may be an important venue for the development of an efficacious adjunctive therapeutic for HAND.
